# Robotic-assisted tendon transplantation—approach for pelvic organ prolapse repair: a case report

**DOI:** 10.1186/s13256-025-05267-2

**Published:** 2025-05-26

**Authors:** Dominique Koensgen, Charlotte Lukannek, Carolin Schroeder, Lucia A. Otten, Amadeus Hornemann, Alexander Mustea

**Affiliations:** 1 Department of Gynecology and Gynecological Oncology, University Hospital Bonn, Venusberg – Campus 1, 5312 Bonn, Germany; 2 Department of Gynecology, Bürgerspital, Frankfurt Am Main, Germany

**Keywords:** Apical prolapse surgery, Tendon transplantation, Robotic-assisted surgery, Case report, Pelvic organ prolapse, Synthetic mesh, Biological graft

## Abstract

**Background:**

The management of pelvic organ prolapse remains a clinical challenge, particularly with concerns regarding the safety of synthetic mesh-based procedures. In response, we present a novel approach for apical prolapse repair utilizing robotic assistance for tendon transplantation, offering a promising alternative to the use of synthetic meshes.

**Case presentation:**

A 47-year-old multiparous woman (white, non-Hispanic) with a history of three vaginal deliveries and laparoscopic supracervical hysterectomy presented with symptoms of pelvic organ prolapse, including vaginal discomfort, recurrent urinary tract infections, and overactive bladder. Clinical examination and ultrasonography revealed a combined anterior and apical vaginal prolapse grade II according to the Pelvic Organ Prolapse Quantification system. A robotic sacrocervicopexy with a semitendinosus autograft, harvested from the left popliteal fossa, and concomitant anterior colporrhaphy were performed. The surgical procedure included the dissection of the ligamentum longitudinale using a nerve-sparing technique, cervix preparation, and tendon transplantation. The surgery lasted 172 min without intraoperative complications. The patient’s postoperative recovery was swift without residual prolapse, voiding dysfunction, or urinary incontinence reported at any of the follow-up examinations, conducted at 8 weeks, 3 months, 6 months, and 1 year after the operation.

**Conclusion:**

This case report demonstrates the successful application of a robotic-assisted tendon transplantation approach for apical prolapse repair, offering a safe and effective alternative to synthetic mesh-based surgical procedures. An ongoing multicenter registry study aims to further validate the method’s safety and efficacy, paving the way for its broader adoption in clinical practice.

## Background

Pelvic organ prolapse is a common condition which affects 40–60% of parous women, describing the descent of one or more of the pelvic organs (uterus, vagina, bladder, or bowel) [1].

It is categorized into various types, including apical vaginal prolapse (involving the uterus or vaginal vault post-hysterectomy), anterior vaginal prolapse (involving cystocele or urethrocele), and posterior vaginal prolapse (involving rectocele or enterocele). The degree of prolapse is often classified using the Pelvic Organ Prolapse Quantification (POP-Q) system, which assigns a specific stage to the extent of prolapse in different compartments of the pelvis. In this system, stages range from 0 (no prolapse) to IV (maximum prolapse) and are used to assess the severity and location of prolapse [2].

The surgical management of apical vaginal prolapse presents various techniques and graft options, spanning vaginal and abdominal approaches performed through open surgery, laparoscopy, or robotic assistance. Studies have shown that abdominal procedures are associated with lower rates of recurrent prolapse, urinary stress incontinence (SUI), and painful intercourse compared with various vaginal surgical procedures [3].

Recent safety concerns surrounding vaginal meshes have led to widespread discussions and regulatory actions by both the FDA (Food and Drug Administration) and NHS (National Health Service). These concerns have prompted a reassessment of the use of synthetic meshes in pelvic organ prolapse surgery, underscoring the need for alternative techniques involving biological grafts.

Hornemann *et al*. introduced a novel approach by employing a semitendinosus tendon autograft, commonly used for anterior cruciate ligament reconstruction, for laparoscopic apical prolapse repair and emphasized the feasibility and efficacy of this innovative procedure [4]. Subsequently, Hornemann *et al*. conducted a prospective clinical feasibility study to explore tendon transplantation for pelvic organ prolapse repair via laparoscopic pectopexy, further confirming the safety and effectiveness of this method using a semitendinosus autograft instead of synthetic mesh [5]. To assess the potential of tendon transplantation in pelvic organ prolapse repair via laparoscopic sacropexy, a multicenter study was initiated, yielding initial results from 20 patients after 6 months of follow-up, which affirmed the method’s safety and efficacy [6]. Meanwhile, an adaptation of the procedure was established, utilizing only half the width of the semitendinosus tendon and thereby reducing leg morbidity [7, 8].

## Case presentation

This case was reported in 2022 in the University Hospital of Bonn.

A 47-year-old gravida 3, para 3, (white, non-Hispanic) with a medical history that included three vaginal deliveries and a laparoscopic supracervical hysterectomy (LASH) for adenomyosis uteri 4 years prior, presented at our department. The patient reported escalating bulge symptoms, vaginal discomfort, recurrent lower urinary tract infections, a 10 pack-year history of smoking, and being slightly overweight with a BMI of 26.7 kg/m^2^. No other significant medical conditions were noted. She had been diagnosed with overactive bladder (OAB dry) 5 years earlier. She had not undergone any conservative treatments such as physical therapy during the 5-year period.

Clinical examination and ultrasonography revealed a combined anterior and apical vaginal prolapse grade II, as per the Pelvic Organ Prolapse Quantification (POP-Q) system, with a central cystocele grade II (anterior compartment) and cervical prolapse grade II (apical compartment).

Following a preoperative urodynamic study, the patient was scheduled for a robotic sacrocervicopexy with a semitendinosus autograft and concomitant anterior colporrhaphy.

**Fig. 1:**
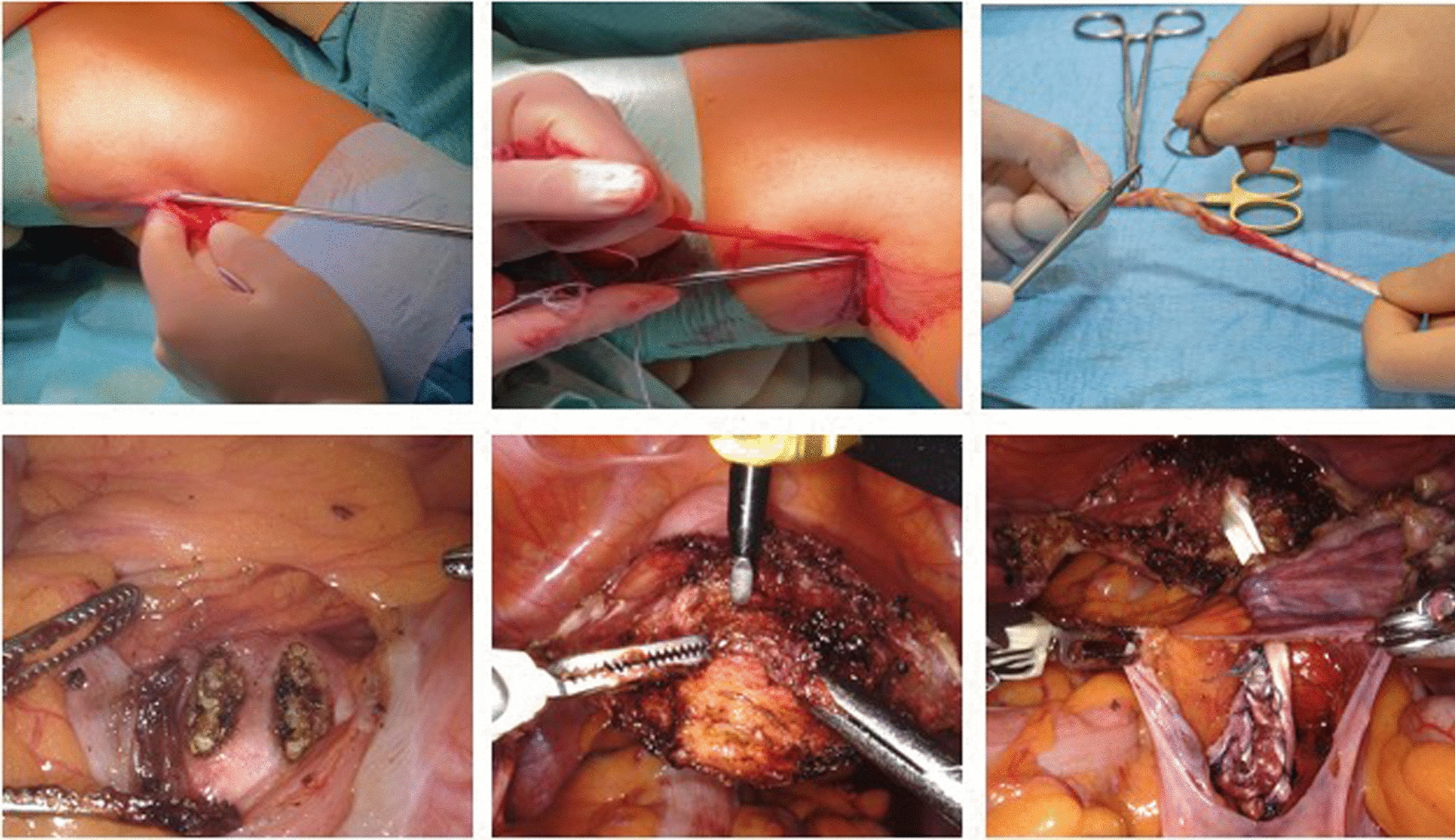
Illustration of the key surgical steps: removing of semitendinosus tendon (**a**, **b**). Preparation of removed tendon (**c**). Incision of ligamentum longitudinale (**d**). Perforation of cervix (**e**). Suturing of tendon (**f**)

The surgery was conducted under general anesthesia. A standard single-shot antibiotic prophylaxis with cefuroxim 1.5 g and metronidazole 0.5 g was administered preoperatively. The semitendinosus tendon was harvested from the popliteal fossa of the patient’s left leg [8]. A 25 mm incision was made in the sterile-draped popliteal cavity, and the fascia covering the semitendinosus tendon was incised. Subsequently, an open 7 mm tendon harvester (Arthrex Inc., Naples, Florida, USA) was placed over the tendon, and by pushing it proximally, this portion of the tendon was separated from the semitendinosus muscle (Fig. 1a, b). The distal part of the tendon was removed from the pes anserinus using a 7 mm closed harvester (Arthrex Inc., Naples, Florida, USA) in the same manner. The incision in the popliteal cavity was closed with two single stitches. Both ends of the removed tendon were prepared with a sling suture (HoTT^®^-Sling) to prevent tendon splitting (Fig. 1c). Subsequently, robotic-assisted daVinci Xi laparoscopy was performed. Three robotic trocars were placed umbilically and 8 cm lateral to each side, with an additional 10 mm assist trocar placed suprasymphysially. A fenestred bipolar clamp and a monopolar scissor were utilized to perform the following surgical steps.

The ligamentum longitudinale was dissected using a nerve-sparing technique. Then the ligamentum longitudinale was incised in the L5/S1 region over a length of 20 mm and at a distance of 10 mm (Fig. 1d). The cervix was then prepared by opening the plica vesico-uterina and moving the bladder away from the cervix. Two perforations were made through the cervix in a ventral to dorsal–cranial direction using a monopolar spatula (CleanCoat TM Laparoscopic Curved 36 cm, Covidien TM) (Fig. 1e). The peritoneum was tunneled on the right side of the pelvis. The prepared tendon was introduced into the abdomen through the suprasymphyseal 10 mm assist trocar. The tendon was pulled through the cervix, passed through the peritoneal tunnel, and pulled through the ligamentum longitudinale. The ends of the tendon were attached to each other with a side-to-side sling suture (HoTT^®^-Sling) (Fig. 1f). The peritoneum was closed with resorbable sutures, and in the final step, an anterior colporrhaphy was performed using resorbable sutures. The total operation time was 172 min, with no intraoperative complications and minimal blood loss of 30 mL.

The patient had a rapid recovery and was discharged on the fourth day after surgery. Clinical examination upon discharge revealed no pelvic organ prolapse in the anterior and apical compartments. Ultrasonography showed no residual urine, and no voiding dysfunction was reported.

At each of the four follow-up visits conducted at 8 weeks, 3 months, 6 months, and 1 year postoperatively, the clinical examination revealed no pathological findings.

Both objective and subjective outcomes were assessed preoperatively and during each follow-up visit using the POP-Q system and the standardized German Pelvic Floor Questionnaire, respectively. There were no signs of pelvic organ prolapse, bladder dysfunction, or bowel dysfunction and no signs of leg dysfunction.

The patient expressed high satisfaction.

## Discussion and conclusions

This case report introduces a novel robotic-assisted approach using a semitendinosus tendon autograft for pelvic organ prolapse repair, offering a promising alternative to synthetic mesh-based procedures. The growing safety concerns around vaginal mesh have prompted the need for safer alternatives, particularly for apical prolapse repair. The successful outcome in this case illustrates the potential of using tendon autografts, which provide biological compatibility and avoid complications associated with synthetic materials.

There is increasing support for non-mesh alternatives in pelvic organ prolapse management. Studies on native tissue repair for posterior compartment prolapse (rectocele) demonstrate significant improvements in anatomical support and functional outcomes, with high patient satisfaction and reduced recurrence rates and mesh related complications. This is particularly relevant for patients who seek alternatives to mesh-based procedures owing to concerns about long-term complications [9].

The innovative use of a tendon autograft in this case aligns with the broader shift towards non-mesh solutions. While tendon autografts may present challenges, such as donor site morbidity, modifications to the procedure (e.g., using only half of the tendon to reduce leg morbidity) suggest that these risks can be minimized [7, 8, 10]. In our case, no leg complications were observed, though formal functional assessments were not performed during the follow-up visits. This highlights the need for further research to assess both the functional impact of tendon harvesting and the long-term efficacy of this approach.

Robotic-assisted tendon transplantation may involve higher initial costs owing to the complexity of the procedure and robotic equipment. However, the use of the patient’s own tendon eliminates the cost of synthetic mesh materials. In addition, when considering the avoidance of mesh-related complications and potential reoperations, this approach may offer long-term cost savings. Future comparative studies examining operative time, costs, and patient-reported outcomes for both mesh and non-mesh procedures will be essential to validate these findings.

In addition, as this case involved robotic surgery, the discussion of operative techniques must also acknowledge advancements in surgical technologies. Robotic surgery, known for improved dexterity and precision, likely contributed to the favorable outcomes observed in this patient. As evidence on robotic and laparoscopic procedures continues to grow, it is important to contextualize these techniques within the broader spectrum of native tissue and synthetic approaches.

In conclusion, the use of robotic-assisted tendon transplantation for POP repair offers a viable alternative to mesh-based procedures, addressing the growing demand for safer, effective surgical options in the field. The promising short-term results from this case, combined with the broader evidence supporting non-mesh solutions in pelvic organ prolapse surgery, underscore the potential of this innovative approach [8]. Ongoing studies, including multicenter trials, will be critical in determining the long-term efficacy and cost-effectiveness of these techniques.

## Data Availability

Not applicable.
